# Effects of a mobile app-based biofeedback breathing exercise program on handgrip strength, respiratory muscle activity, and pulmonary function in healthy adults

**DOI:** 10.3389/fresc.2025.1696503

**Published:** 2025-12-18

**Authors:** Tae-Woo Kang, Seo-Yoon Park, Hee-Jin Jo

**Affiliations:** 1 Department of Physical Therapy, Woosuk University, Jeonju, Republic of Korea; 2 Graduate Program in Physical Therapy, Woosuk University, Jeonju, Republic of Korea

**Keywords:** breathing exercise, electromyography, handgrip strength, mobile application, pulmonary function, rehabilitation, respiratory efficiency, young adults

## Abstract

**Introduction:**

This study investigated the effects of a mobile app–based biofeedback breathing exercise program on handgrip strength, respiratory muscle activity, and pulmonary function in healthy young adults.

**Methods:**

Forty-eight participants were randomly assigned to an experimental group (*n* = 22), which performed app-based biofeedback breathing exercises, or a control group (*n* = 26), which performed traditional breathing exercises. Both groups completed three to four sessions per week for four weeks. Handgrip strength, respiratory muscle activity measured using surface electromyography, and pulmonary function assessed by spirometry were evaluated before and after the intervention.

**Results:**

The experimental group showed a significant within-group improvement in handgrip strength (*p* < 0.05), although between-group differences were not significant. Significant reductions in external intercostal and rectus abdominis activity were observed in the experimental group (*p* < 0.05), whereas the control group demonstrated reduced external oblique activity (*p* < 0.05). Both groups improved in forced expiratory volume in one second (FEV₁) and in the FEV₁/forced vital capacity (FVC) ratio (*p* < 0.05), with a greater improvement in FEV₁/FVC observed in the experimental group.

**Discussion:**

These findings suggest that mobile app–based biofeedback breathing exercises may enhance respiratory efficiency and optimize muscle activation patterns in healthy young adults, supporting their potential use as an accessible adjunct tool in pulmonary rehabilitation.

## 1 Introduction

Breathing is a fundamental physiological process essential for human survival, carried out through repeated cycles of inhaling oxygen and exhaling carbon dioxide. Various training methods, such as inspiratory muscle training and resistance-based exercises, have been developed to enhance respiratory efficiency, showing positive effects on core stability and physical endurance (1, 2). However, maintaining consistent rhythm, intensity, and engagement during these exercises can be challenging, highlighting the need for structured and motivating approaches (3).

Respiratory mechanics primarily rely on the diaphragm and thoracic structures. The diaphragm, assisted by the intercostal muscles, serves as the primary driver of ventilation (4). Reduced diaphragmatic efficiency can increase reliance on accessory muscles, which may lead to less efficient breathing (5). Moreover, poor posture and weakened musculature may further impair respiratory function, increasing susceptibility to respiratory conditions such as asthma or pneumonia. Thoracic mobility is equally critical, as restricted chest expansion limits lung capacity and effective breathing (6). Interventions targeting thoracic flexibility and muscle efficiency have demonstrated benefits in both healthy individuals and patients undergoing cardiopulmonary rehabilitation (7).

Traditionally, breathing training has been guided by health professionals; however, the growing demand for technology-driven and cost-effective approaches has led to the development of mobile health applications (8). A wide variety of mobile apps specifically designed for breathing exercises are now available, while breathing modules are also frequently integrated into stress and anxiety management applications (9–11). Most of these programs employ visual or auditory pacing cues to guide respiratory rhythm, and studies have shown that wave-based visualizations of breathing patterns are easier to follow than circle-based or purely auditory feedback formats (12)

Despite growing evidence supporting the benefits of mobile health–based interventions, research on the physiological effects of mobile app–based biofeedback breathing exercises—particularly regarding respiratory muscle activation, pulmonary function, and physical performance in healthy young adults—remains limited. Additionally, handgrip strength was included as a primary outcome because recent evidence has demonstrated a positive causal association between pulmonary function parameters (such as FVC and FEV₁) and grip strength (13). This suggests that breathing-related physiological improvements may also be reflected in functional strength performance, supporting the relevance of evaluating handgrip strength in this study. Therefore, the present study aimed to investigate the effects of a mobile app–based biofeedback breathing exercise program compared with traditional breathing exercises on handgrip strength, respiratory muscle activity, and pulmonary function. By exploring these outcomes, this study seeks to provide preliminary evidence for the potential applicability of mobile app–assisted breathing training as a practical and accessible tool for respiratory rehabilitation and wellness promotion.

## 2 Materials and methods

### 2.1 Participants

Participants were recruited between June 1 and September 30, 2024, through offline bulletin board announcements at Woosuk University. A total of 52 healthy adults voluntarily enrolled after receiving information about the study's purpose and procedures. All data collection procedures, including baseline and post-intervention assessments, were conducted at the Human Performance Laboratory of Woosuk University in Jeonju, South Korea. The required sample size was calculated using G*Power with a significance level (*α* = 0.05), statistical power (1–*β* = 0.80), and an expected large effect size (Cohen's d = 0.8), based on prior respiratory training studies reporting medium-to-large improvements in pulmonary function and respiratory muscle activation. This indicated that at least 21 participants were needed per group. To account for a predicted dropout rate of approximately 19%, 26 participants were initially recruited for each group. Inclusion criteria were: (1) age ≥ 20 years, (2) ability to communicate effectively, (3) absence of auditory or visual impairments, and (4) voluntary agreement to participate. Exclusion criteria were: (1) musculoskeletal conditions interfering with participation in breathing exercises, or (2) use of medications that could affect respiratory function. This study was approved by the Institutional Bioethics Committee of Woosuk University (approval no. WS-2024-42; approval date: March 18, 2024). All participants provided written informed consent prior to enrollment. Randomization was performed using a computer-generated allocation list, and allocation concealment was maintained using sequentially numbered, opaque, sealed envelopes. Outcome assessors and data analysts were blinded to group assignment. The random allocation sequence was generated by an investigator not involved in participant recruitment or data collection. A separate research assistant enrolled participants, and an independent administrator assigned participants to each group using the sealed envelopes. The primary outcome was defined *a priori* as FEV₁ measured at post-test, while secondary outcomes included FEV₁/FVC, FVC, respiratory muscle activity (sEMG), and handgrip strength. This study was not preregistered, and this has been stated transparently.

### 2.2 Intervention

**Figure 1:**
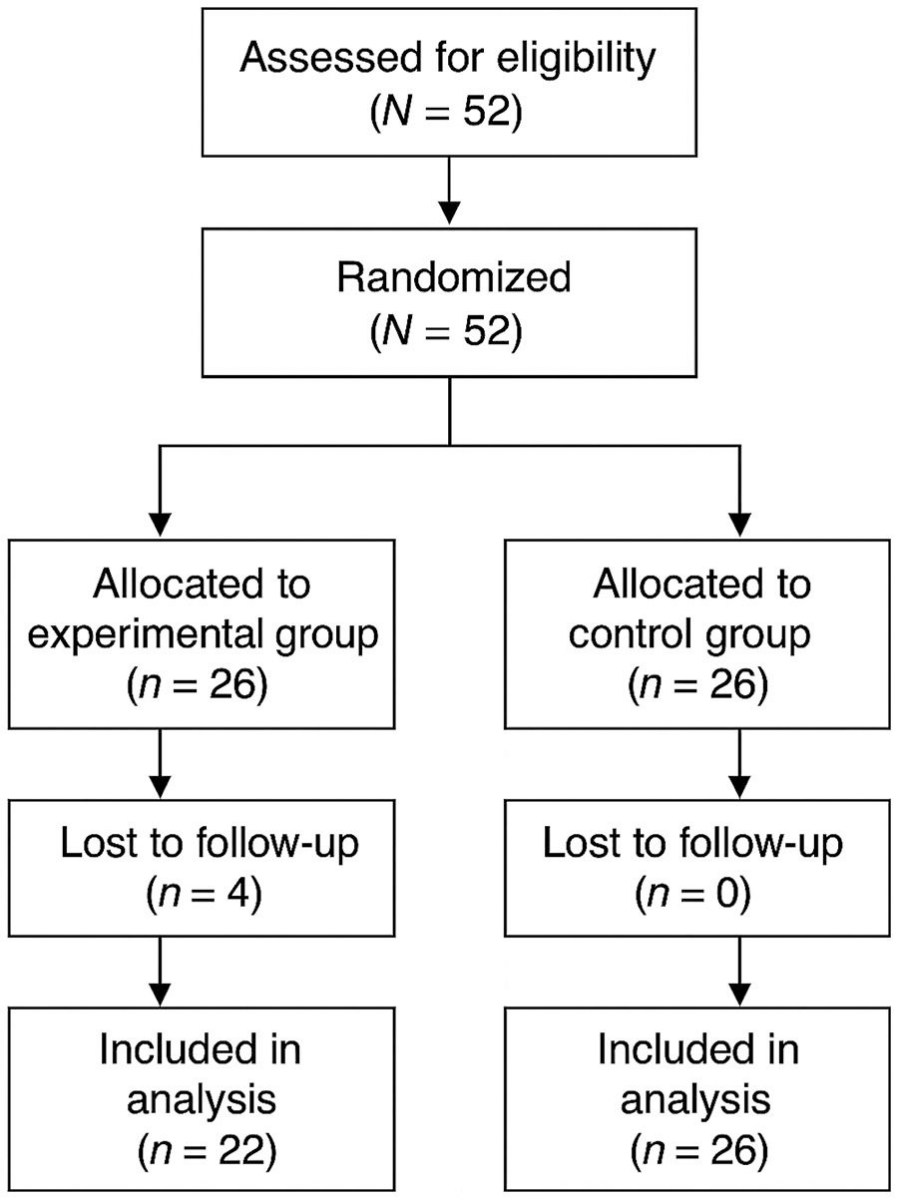
CONSORT flow diagram of participant enrollment, allocation, intervention, and analysis.

This study employed a randomized pretest–posttest design. Participants were randomly assigned to either an experimental group (EG) or a control group (CG). Initially, 26 participants were allocated to each group; however, four participants withdrew from the EG during the study due to schedule conflicts and personal reasons unrelated to the intervention, resulting in final sample sizes of EG (*n* = 22) and CG (*n* = 26). The participant flow throughout enrollment, allocation, follow-up, and analysis is summarized in the CONSORT flow diagram (Figure 1).

**Figure 2:**
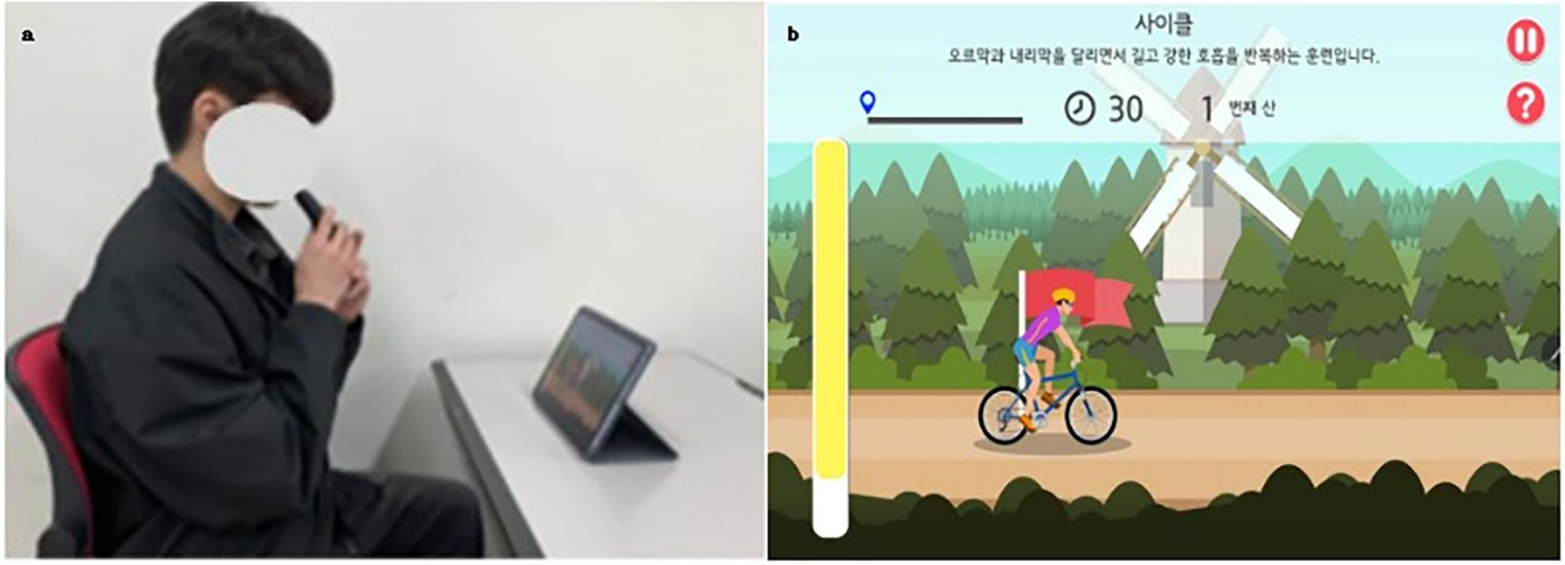
**(a)** breathing exercise using the “Voice on” application; **(b)** cycling game module in the “Voice on” application.

Participants in the experimental group performed mobile app–based biofeedback breathing exercises using the Breathe-On device (HUWANT, Korea), which was connected to a smartphone running the Voice On application. In the app's *Cycling* module, participants generated expiratory airflow to propel a digital cyclist forward, receiving continuous real-time visual biofeedback (Figure 2). Each session consisted of three 2-minute breathing bouts, each separated by 1-minute rest intervals, for a total of approximately 8 min. During the exercise, participants followed the paced rhythm displayed on-screen, typically inhaling for approximately 2–3 s and exhaling for approximately 4–5 s. Minor individual variations occurred, but the program required sustained expiratory effort to advance the module, resulting in a consistently exhalation-dominant breathing patternacross participants. Training sessions were conducted three times per week for four weeks. This paced, exhalation-dominant breathing pattern is consistent with established physiological evidence demonstrating that slow, controlled breathing enhances autonomic regulation and respiratory efficiency (14).

**Figure 3:**
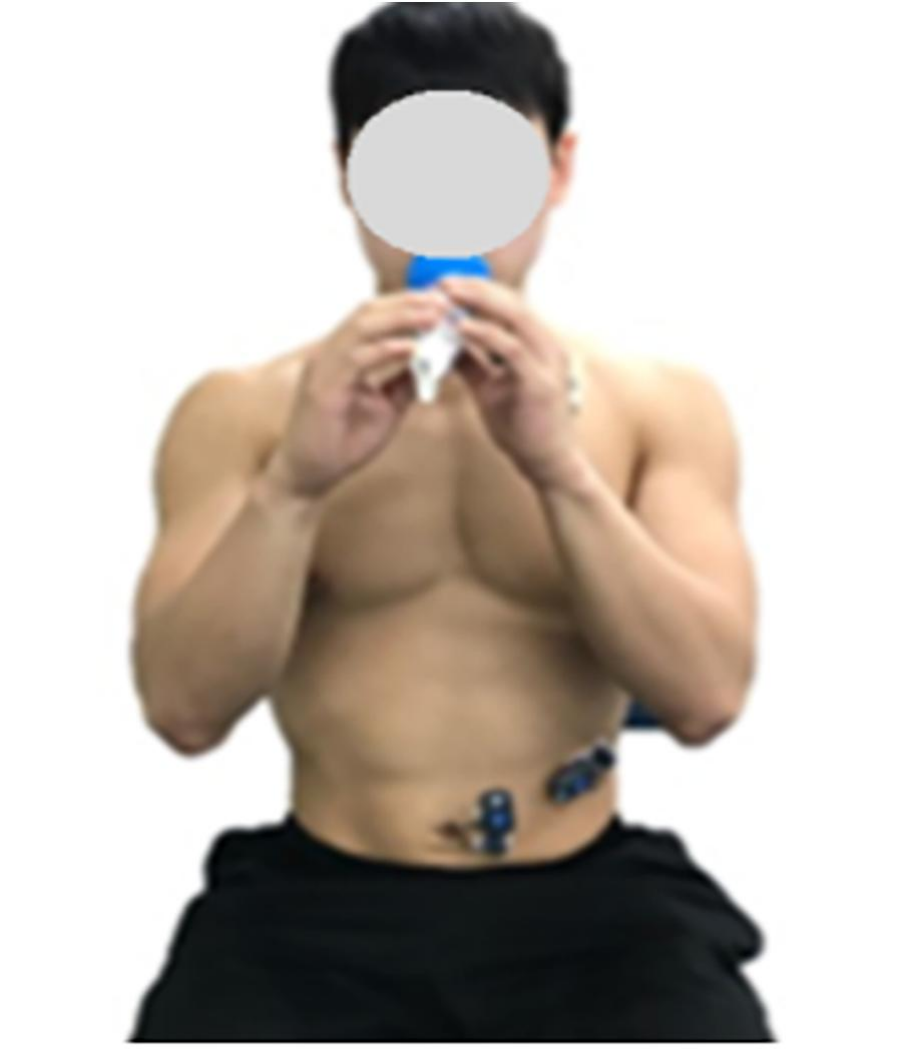
Breathing exercise protocol using the “Big Breathe” device.

Participants in the control group used the Big-Breathe device (HUWANT, Korea) for traditional resistance-based inspiratory muscle training (Figure 3). Each session consisted of five sets, each including four 5-second inhalations, followed by relaxed exhalation lasting approximately 4–5 s, paced verbally by the examiner. Thirty-second rest intervals were provided between sets, and the total session duration was approximately 3 min and 40 s. Although the training frequency and program duration (three times per week for four weeks) were matched between groups, the session duration differed, with the experimental group training for approximately 8 min compared to 3 min and 40 s in the control group. This discrepancy in training dose is acknowledged as a potential confounding factor. Attendance was recorded for all participants, and in-app usage logs were monitored to verify adherence in the experimental group. This resistance-based breathing protocol is in line with established respiratory muscle training parameters supported by meta-analytic evidence in healthy adults (15).

### 2.3 Measurements

#### 2.3.1 Hand grip strength

Handgrip strength was measured using a hydraulic hand dynamometer (Saehan Corp., Masan, Korea). The device has demonstrated excellent reliability [intraclass correlation coefficient (ICC) = 0.90–0.98] (16) and validity (r = 0.85–0.95) (17). Participants were seated with their shoulders adducted and neutrally rotated, elbows flexed at 90°, and forearms in a neutral position. Each hand was tested three times. Although both hands were assessed, the mean value of the dominant hand (average of three trials) was used for statistical analysis to reduce inter-limb variability and in accordance with common handgrip testing practices.

#### 2.3.2 Respiratory muscle activation

Respiratory muscle activation was assessed using surface electromyography (sEMG) with the BTS FreeEMG 1000 system (BTS Bioengineering, Italy), which demonstrates high reliability (ICC = 0.83–0.94) (18) and validity (*r* = 0.85–0.94) (19). Electrodes were placed over the sternocleidomastoid, external intercostal, external oblique, and rectus abdominis muscles. Ten natural breathing cycles were recorded in a seated position, excluding the first and last two cycles. Root mean square (RMS) values during maximal inspiratory effort, performed using the Big Breathe device, were used for normalization. Muscle activity was expressed as a percentage of the reference voluntary contraction (%RVC).

#### 2.3.3 Pulmonary function

Pulmonary function was measured using a spirometer (Pony Fx, Cosmed, Italy), which demonstrates excellent reliability (ICC = 0.99) and validity (*r* = 0.85–0.95) (20, 21). Participants were seated with hips and knees flexed at 90° and instructed to perform three trials of maximal inhalation followed by forced exhalation. The average of the three trials was used for analysis. The following parameters were recorded: forced vital capacity (FVC), forced expiratory volume in one second (FEV₁), and the FEV₁/forced vital capacity (FVC) ratio.

### 2.4 Statistical analysis

All statistical analyses were performed using SPSS version 22.0 (IBM Corp., Armonk, NY, USA). Descriptive statistics were used to summarize participant demographics, and the Kolmogorov–Smirnov test was applied to assess data normality. Within-group pre–post differences were analyzed using paired *t*-tests, and between-group comparisons were performed using independent *t*-tests. This approach was selected due to the small sample size and balanced randomization, which minimized baseline imbalance. To enhance interpretability beyond *p*-values, standardized effect sizes (Cohen's d) and their 95% confidence intervals were calculated for all primary and secondary outcomes. This study used a per-protocol (complete-case) analysis, as all participants who completed the intervention also completed all outcome assessments. No missing data were present, and therefore no imputation procedures were required and an intention-to-treat (ITT) analysis was not applicable. All statistical tests were two-tailed with the significance level set at *p* < 0.05. Although more complex models (e.g., mixed ANOVA or ANCOVA) can provide additional adjustments, the current design and sample size justified the use of *t*-tests as an appropriate and transparent method for this exploratory pilot trial.

## 3 Results

### 3.1 General characteristics of participants

A total of 52 healthy young adults in their twenties were recruited for the study. The required sample size was calculated using G*Power (version 3.1) with *α* = 0.05, power = 0.80, and an effect size of *d* = 0.8, indicating a minimum of 21 participants per group. To account for an anticipated dropout rate of approximately 19%, 52 participants were enrolled and randomly assigned to the experimental group (EG, *n* = 26) or the control group (CG, *n* = 26). During the intervention period, four participants in the EG withdrew, resulting in final sample sizes of EG (*n* = 22) and CG (*n* = 26) (Figure 1). Baseline characteristics—including sex distribution, age, height, weight, and body mass index (BMI)—did not significantly differ between groups (*p* > 0.05), confirming initial homogeneity (Table 1).

**Table 1:** General characteristics of participants in the experimental and control groups.

Characteristics	Experimental group (*n* = 22) Mean ± SD	Control Group (*n* = 26) Mean ± SD	*χ*2/t	p
Age (years)	25.27 ± 0.88	25.08 ± 1.01	−0.076	0.484
Height (cm)	169.2 ± 6.62	170.8 ± 6.15	0.846	0.402
Body weight (kg)	65.70 ± 14.25	72.42 ± 10.96	1.843	0.072
Gender (male/female)	10/12	9/17	0.585	0.444
BMI (kg/m2)	22.96	24.92	1.982	0.053

SD, standard deviation; BMI, body mass index.

### 3.2 Hand grip strength

Hand grip strength outcomes are presented in Table 2. In the experimental group (EG), mean handgrip strength increased significantly from 36.42 ± 9.70 kg to 37.25 ± 10.17 kg (t = –3.364, *p* = 0.003, d = 0.47). In the control group (CG), handgrip strength increased slightly from 37.68 ± 8.77 kg to 38.00 ± 9.91 kg, but this change was not statistically significant (t = –0.327, *p* = 0.746, d = 0.06). Between-group comparisons revealed no significant differences at baseline (t = 0.470, *p* = 0.641) or post-test (t = 0.260, *p* = 0.796). The mean change scores were 0.83 ± 2.33 kg in the EG and 0.32 ± 1.99 kg in the CG. Both groups' changes were below the commonly reported MDC₉₅ of approximately 5.0–6.5 kg for handgrip strength and were therefore coded as “No” for exceeding MDC.

**Table 2:** Comparison of hand grip strength between the experimental and control groups.

Measure	Experimental group (*n* = 22) Mean ± SD	Control Group (*n* = 26) Mean ± SD	t	p
Pre-test	36.42 ± 9.79	37.68 ± 8.77	0.470	0.641
Post-test	37.25 ± 10.17	38.00 ± 9.91	0.260	0.796
t	−3.364	−0.327		
P	0.003*	0.746		
Change (Post–Pre)	0.83 ± 1.76	0.32 ± 5.13	0.475	0.638
Effect size (Cohen's d)	0.47	0.06		
Exceeds MDC (5–6 kg)	No	No		

MDC₉₅ for handgrip strength in healthy adults is approximately 5–6 kg (15); therefore, neither group exceeded this threshold.

SD, standard deviation.

* *p* < 0.05.

### 3.3 Respiratory muscle activity

Surface electromyography (sEMG) results are summarized in Table 3. For the sternocleidomastoid (SCM), muscle activity decreased slightly in both groups (EG: 20.76 ± 8.35% → 18.38 ± 11.91%, CG: 30.92 ± 21.24% → 22.93 ± 21.39%), but neither change reached statistical significance (*p* > 0.05). The effect sizes were small in both groups (EG: d = –0.20, CG: d = –0.30), and no significant between-group difference was observed after the intervention (*p* = 0.335). For the external intercostalis (EI), the EG demonstrated a significant reduction from 46.90 ± 26.84% to 20.43 ± 14.14% (t = 4.274, *p* < 0.001), with a large effect size (d = –0.91). In contrast, the CG showed no meaningful change (t = –0.044, *p* = 0.966; d = –0.01). A significant between-group difference was detected post-intervention (*p* = 0.012*). For the external oblique (EO), activity in the EG showed a marginal decrease (59.89 ± 25.84% → 44.67 ± 18.14%, *p* = 0.058; d = –0.43). The CG exhibited a significant decrease (67.34 ± 31.06% → 49.97 ± 18.92%, *p* = 0.001; d = –0.70). However, no significant between-group difference was found after the intervention (*p* = 0.813). Finally, the rectus abdominis (RA) showed a substantial reduction in the EG (85.88 ± 25.46% → 31.85 ± 26.00%, *p* < 0.001; d = –1.59), whereas the CG displayed a non-significant decrease (94.75 ± 34.85% → 84.30 ± 33.83%, *p* = 0.285; d = –0.21). A significant between-group difference was observed post-intervention (*p* = 0.00073**), indicating a stronger reduction in the EG.

**Table 3:** Comparison of respiratory muscle activity between the experimental and control groups.

Muscle	Test	Experimental group (*n* = 22) Mean ± SD	Control Group (*n* = 26) Mean ± SD	t	p
SCM	pre	20.76 ± 8.35	30.92 ± 21.24	2.108	0.041*
	post	18.38 ± 11.91	22.93 ± 21.39	0.888	0.379
	t	0.932	1.553		
	p	0.362	0.133		
	Change (Post–Pre)	−2.38 ± 11.96	−7.99 ± 26.24	0.977	0.335
	Effect size (Cohen's d)	−0.20	−0.30		
EI	pre	46.90 ± 26.84	35.98 ± 23.64	−1.499	0.141
	post	20.43 ± 14.14	36.17 ± 24.83	2.632	0.012*
	t	4.274	−0.044		
	p	< 0.001**	0.966		
	Change (Post–Pre)	−26.47 ± 29.05	0.19 ± 22.80	−3.490	0.0012**
	Effect size (Cohen's d)	−0.91	−0.01		
EO	pre	59.89 ± 25.84	67.34 ± 31.06	0.898	0.376
	post	44.67 ± 18.14	49.97 ± 18.92	0.985	0.330
	t	2.005	3.581		
	p	0.058	0.001**		
	Change (Post -Pre)	−15.22 ± 35.60	−17.37 ± 24.74	0.239	0.813
	Effect size (Cohen's d)	−0.43	−0.70		
RA	pre	85.88 ± 25.46	94.75 ± 34.85	0.990	0.327
	post	31.85 ± 26.00	84.30 ± 33.83	5.820	<0.001**
	t	7.439	1.092		
	p	< 0.001**	0.285		
	Change (Post -Pre)	−54.02 ± 34.06	−10.45 ± 48.77	−3.628	0.00073**
	Effect size (Cohen's d)	−1.59	−0.21		

SCM, sternocleidomastoid; EI, external intercostal; EO, external oblique; RA, rectus abdominis; SD, standard deviation.

* *p* < 0.05, **p < 0.01.

### 3.4 Pulmonary function

Pulmonary function outcomes are summarized in Table 4. For FVC, the EG showed a slight decrease from 3.78 ± 0.27 L to 3.75 ± 0.20 L (t = 0.353, *p* = 0.727, d = –0.08), whereas the CG showed a small increase from 3.93 ± 0.69 L to 3.95 ± 0.67 L (t = –0.407, *p* = 0.688, d = 0.08). The between-group comparison revealed no significant differences at baseline (t = 0.978, *p* = 0.333) or post-test (t = 1.327, *p* = 0.191). Both groups were coded as “N/A” for MCID/MDC because no widely applicable thresholds exist for healthy adults. For FEV₁, the EG increased significantly from 2.91 ± 0.29 L to 3.32 ± 0.32 L (t = –4.557, *p* < 0.001, d = 0.97), and the CG also showed a significant increase from 2.89 ± 0.71 L to 3.25 ± 0.70 L (t = –3.437, *p* = 0.002, d = 0.67). No significant between-group difference was observed after the intervention (t = –0.412, *p* = 0.341). MCID/MDC was coded as “N/A” due to the absence of validated thresholds for non-disease populations. For FEV₁/FVC (%), the EG improved significantly from 76.77 ± 8.41% to 87.00 ± 6.55% (t = –4.053, *p* < 0.001, d = 0.86), and the CG improved from 73.27 ± 14.72% to 81.58 ± 9.79% (t = –3.889, *p* < 0.001, d = 0.76). A significant between-group difference was observed post-intervention (t = –2.211, *p* = 0.032), favoring the EG. As with the other variables, MCID/MDC was coded as “N/A.”

**Table 4:** Comparison of pulmonary function between the experimental and control groups.

Measure	Test	Experimental group (*n* = 22) Mean ± SD	Control Group (*n* = 26) Mean ± SD	t	p
FVC (L)	Pre-test	3.78 ± 0.27	3.93 ± 0.69	0.978	0.333
	Post-test	3.75 ± 0.20	3.95 ± 0.67	1.327	0.191
	t	0.353	−0.407		
	p	0.727	0.688		
	Change (Post–Pre)	−0.03 ± 0.34	0.02 ± 0.22	−0.611	0.544
	Effect size (Cohen's d)	−0.08	0.08		
	Exceeds MCID/MDC	N/A	N/A		
FEV1 (L)	pre	2.91 ± 0.29	2.89 ± 0.71	−0.141	0.444
	post	3.32 ± 0.32	3.25 ± 0.70	−0.412	0.341
	t	−4.557	−3.437		
	p	<0.001**	0.002*		
	Change (Post–Pre)	0.41 ± 0.42	0.37 ± 0.54	0.291	0.773
	Effect size (Cohen's d)	0.97	0.67		
	Exceeds MCID/MDC	N/A	N/A		
FEV1/ FVC (%)	pre	76.77 ± 8.41	73.27 ± 14.72	−0.987	0.329
	post	87.00 ± 6.55	81.58 ± 9.79	−2.211	0.032*
	t	−4.053	−3.889		
	p	<0.001**	<0.001**		
	Change (Post–Pre)	10.23 ± 11.84	8.31 ± 10.89	0.605	0.548
	Effect size (Cohen's d)	0.86	0.76		
	Exceeds MCID/MDC	N/A	N/A		

FVC, forced vital capacity; FEV1, forced expiratory volume in 1 s; SD, standard deviation.

Exceeds MCID/MDC: Existing MCID/MDC thresholds for pulmonary function measures (e.g., FVC, FEV₁, FEV₁/FVC%) have been established primarily in disease-specific populations, such as patients with idiopathic pulmonary fibrosis (IPF) (26). Because no widely applicable thresholds are available for healthy adults or non-pulmonary clinical populations, MCID/MDC criteria were not applied in the present study, and all outcomes were coded as “N/A.”.

* *p* < 0.05, **p < 0.01.

## 4 Discussion

This study examined the effects of a mobile app–based biofeedback breathing exercise program on handgrip strength (HGS), respiratory muscle activity, and pulmonary function in healthy young adults. The main findings were as follows: (1) HGS significantly improved in the experimental group (EG); (2) external intercostal (EI) and rectus abdominis (RA) activity decreased in the EG, indicating reduced activation of accessory respiratory muscles during breathing; and (3) significant within-group improvements were observed in FEV₁ and the FEV₁/FVC ratio, with a post-intervention between-group difference noted only for the FEV₁/FVC ratio. Collectively, these findings suggest that structured, feedback-guided breathing training delivered through a mobile app may contribute to improved neuromuscular control during breathing and certain aspects of pulmonary function in healthy individuals.

### 4.1 Hand grip strength

A significant increase in hand grip strength (HGS) was observed in the experimental group (EG), whereas no comparable change occurred in the control group (CG). Although the breathing intervention did not directly target upper-limb musculature, repeated, feedback-guided respiratory efforts may have influenced neuromuscular engagement beyond the primary respiratory muscles. Previous studies have reported that inspiratory muscle training and controlled breathing tasks can affect trunk muscle activation and postural control mechanisms (1–3, 13, 22). Because trunk stabilization and respiratory–postural interactions contribute to efficient force transmission during gripping tasks (4–6), such mechanisms may partly relate to the HGS improvement observed in the present study. However, as trunk muscle activity and intra-abdominal pressure were not directly measured, these interpretations should be considered cautiously. Overall, the findings suggest that breathing-focused training may have secondary effects on functional strength measures such as HGS in healthy individuals.

### 4.2 Respiratory muscle activity

Significant reductions in external intercostal (EI) and rectus abdominis (RA) activity were observed in the experimental group (EG) following the intervention. Although the present study did not directly assess diaphragmatic motion or respiratory mechanics, decreased activation of these accessory muscles may reflect altered neuromuscular recruitment during breathing. Prior work has shown that feedback-driven breathing interventions can influence respiratory motor patterns and reduce excessive activation of non-primary respiratory muscles (3, 23). The biofeedback-based mobile app used in this study may have encouraged participants to regulate their breathing rhythm and depth, facilitating more controlled respiratory patterns. In contrast, sternocleidomastoid (SCM) and external oblique (EO) activity did not significantly change, which may be related to variability in individual breathing habits and the time required to modify long-standing accessory muscle behaviors (24). reported that extended training durations are often necessary to alter deeply ingrained respiratory patterns. Taken together, these findings suggest that mobile biofeedback breathing programs may help modify respiratory muscle activation strategies, although further research including direct assessment of diaphragmatic function is warranted.

### 4.3 Pulmonary function

Both groups demonstrated improvements in pulmonary function following the intervention, with significant within-group increases in FEV₁ and the FEV₁/FVC ratio. A between-group difference was observed only in the FEV₁/FVC ratio, favoring the experimental group (EG). Although the present study did not directly assess respiratory mechanics, these patterns may reflect changes in the regulation of breathing rather than alterations in static lung volumes, as FVC did not significantly change in either group. Previous research has shown that structured breathing interventions—including inspiratory muscle training and thoracic mobility–based techniques—can influence ventilatory performance and respiratory muscle function (18, 22, 25, 26). The visual pacing and real-time feedback provided by the mobile app may have supported more controlled and consistent respiratory patterns during training, potentially contributing to the observed improvements. However, as airflow dynamics and muscle coordination were not directly measured, these interpretations should be considered cautiously. Overall, the findings suggest that biofeedback-based breathing exercises delivered through a mobile platform may help enhance certain functional aspects of pulmonary performance even in healthy individuals.

### 4.4 Clinical implications

Compared with conventional breathing exercises, mobile app–based biofeedback programs offer an accessible and user-centered approach to respiratory training. By providing visual pacing cues, feedback on breathing depth, and performance tracking, these tools may enhance user engagement and adherence—factors that often limit the effectiveness of traditional home-based breathing programs (8–12). The digital format also enables integration with wearable sensors and remote monitoring systems, which could support personalized breathing interventions outside clinical settings. Although these features suggest potential utility in preventive health management and rehabilitation contexts, the present findings are based on healthy young adults, and long-term physiological effects were not assessed. Therefore, broader clinical applications should be approached cautiously and require further validation in clinical populations and longitudinal designs.

### 4.5 Limitations and future directions

This study has several limitations that should be considered when interpreting the findings. First, the four-week intervention period may not have been sufficient to capture long-term adaptations in respiratory muscle function or pulmonary outcomes. Second, because all participants were healthy young adults, the generalizability of the results to older adults or clinical populations is limited. Given the homogeneity of the sample, handgrip analyses were not adjusted for sex, body size, or physical activity. Third, the accuracy of breathing feedback may have been influenced by variability in smartphone sensor sensitivity, device positioning, and participant adherence during home practice. Although both groups followed the same scheduled training dose, small differences in actual engagement time may still have occurred; therefore, dose-related confounding cannot be ruled out. Additionally, as the study employed a single commercially available mobile application, the observed effects may not represent those of other app-based feedback systems with different algorithms or interface designs. Furthermore, widely accepted MCID or MDC thresholds for pulmonary function measures in healthy adults are not established, which limits the clinical interpretation of changes in FVC, FEV₁, and FEV₁/FVC%. Future research should incorporate longer intervention durations, more diverse demographic or clinical samples, and objective physiological measures—such as diaphragm ultrasound, respiratory kinematics, or metabolic cost analysis—to clarify the mechanisms underlying app-based breathing training. Comparative studies examining different feedback modalities (e.g., visual pacing alone vs. integrated biofeedback) may also help determine which features most effectively support respiratory improvements and user engagement. Longitudinal trials evaluating the persistence of training effects and adherence after program completion would further strengthen the clinical relevance and scalability of mobile biofeedback breathing interventions. Finally, because multiple outcomes were examined, the potential for Type I error inflation cannot be ruled out. Given the exploratory nature and limited sample size of this pilot trial, no formal multiplicity correction (e.g., hierarchical testing or FDR) was applied. A sensitivity analysis adjusting for attendance or session duration was not feasible because exposure time varied minimally across participants. Future adequately powered randomized trials should incorporate multiplicity-control procedures.

## Data Availability

The original contributions presented in the study are included in the article/Supplementary Material, further inquiries can be directed to the corresponding author.
